# Paraquat poisoning by skin absorption: Two case reports and a literature review

**DOI:** 10.3892/etm.2013.1320

**Published:** 2013-09-27

**Authors:** QIAN ZHOU, BAOTIAN KAN, XIANGDONG JIAN, WEI ZHANG, HUIMIN LIU, ZHONGCHEN ZHANG

**Affiliations:** Department of Poisoning and Occupational Disease, Qilu Hospital of Shandong University, Jinan, Shandong 250012, P.R. China

**Keywords:** paraquat, skin injury, poisoning, methylprednisolone

## Abstract

The present report describes two cases of paraquat poisoning by skin absorption. The cases involved contractual workers who were spraying paraquat in an orchard. Whilst spraying, some solution adhered to their skin. The skin developed erythema followed by blistering and hemorrhaging hemorrhagic diabrosis. Six days later the patients were admitted to the Department of Poisoning and Occupational Disease, Qilu Hospital of Shandong University (Jinan, China) with 3 and 2% total body surface area (TBSA) burns, respectively. Surgical debridement was performed and immunosuppressants were administered during the patients’ treatment. The patients were treated successfully and had made a complete recovery following 21 days. From these cases it was examined how paraquat may cause skin injuries and occasionally poisoning. To the best of our knowledge, cases of paraquat poisoning are rare in China. A review of the relevant literature was performed.

## Introduction

Paraquat, a member of the bipyridyl group of herbicides, has been demonstrated to be an effective weedkiller and is used worldwide. When used as intended, the compound is relatively safe; however, misuse has led to a significant number of deaths (1). The herbicide properties of paraquat were identified in 1955 and it was introduced commercially in 1962 (2). Paraquat is inactive when in contact with almost all naturally occurring soils, and is a relatively non-selective foliage-applied contact herbicide (3). The active ingredient in paraquat is 1,1′-dimethyl-4,4′-bipyridinium, a non-volatile, white, crystalline solid, which decomposes at 300°C. 1,1′-Dimethyl-4,4′-bipyridinium is particularly soluble in water, although insoluble in the majority of organic solvents. It is highly toxic for humans, and has been the cause of numerous cases of acute poisoning (4). Pulmonary fibrosis due to lipid peroxidation is a major symptom of paraquat intoxication (5). The majority of cases of paraquat poisoning result from its intentional ingestion, and in moderate to severe cases the cause of death is normally hypoxemia, secondary to lung fibrosis (6). However, paraquat may be absorbed through skin injuries, and since 1978 there have been several reported cases of severe paraquat poisoning by this pathway (7–10). Since the beginning of its widespread use in 2000, acute paraquat poisoning has continued to be a major public health problem in the rural areas of China, normally from deliberate ingestion or accidental exposure (11). To the best of our knowledge, these instances are rare in China. The aim of this case report was to describe the successful treatment of two patients admitted with dermal paraquat absorption following a crop-dusting accident in which they obtained 3 and 2% total body surface area (TBSA) burns, respectively. Informed consent was obtained from the patients. Furthermore, the relevant literature was analyzed and the treatment that led to the successful result was discussed.

## Case reports

### Case 1

**Figure 1:**
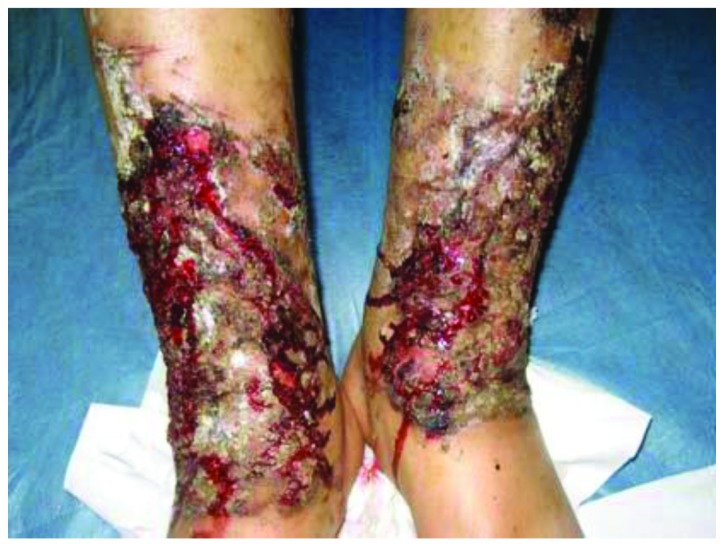
Skin injury caused by paraquat in Case 1.

**Figure 2:**
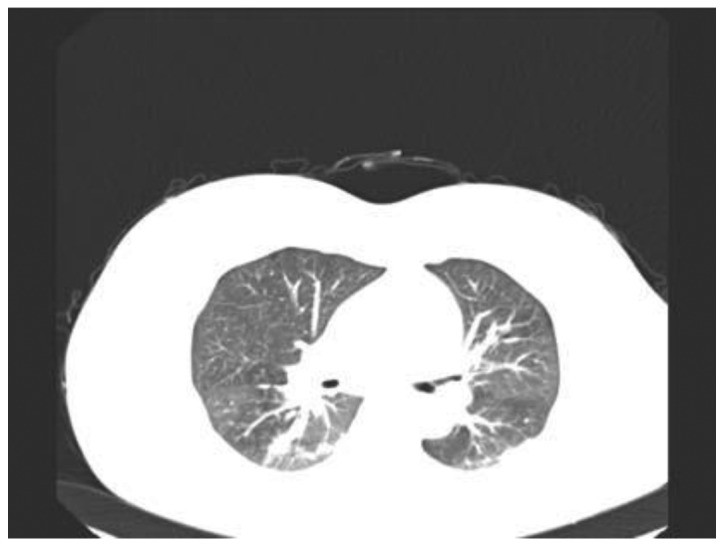
Lung computed tomography (CT) revealed interstitial pneumonitis in Case 1.

A 51-year-old female presented at the Department of Poisoning and Occupational Disease, Qilu Hospital of Shandong University (Jinan, China) with mixed thickness burns to the upper and lower limbs, and with progressive dyspnea. The patient was a contractual worker who had been spraying paraquat in an orchard. The solution in the knapsack spraying device contained 150 ml commercialized concentrated formulation of 20% w/v paraquat and ~50 kg water. The patient had worked from 9:00 am to 12:00 am in the summer and, due to the high temperature (~30°C), the patient’s upper and lower limbs had been fully exposed to the solution, without any protection. The solution had adhered to the patient’s skin, particularly on the lower limbs. The patient did not wash the affected area immediately. In the afternoon, the skin covered by paraquat had developed erythema followed by blistering and hemorrhaging hemorrhagic diabrosis. The patient was treated by a community doctor for five days. Six days subsequent to the incident, the patient’s condition deteriorated, with progressive dyspnea, and the patient was sent to the Qilu Hospital of Shandong University. Upon admission a general examination revealed that the mentation of the patient was intact and the mental status was normal. Physically, the patient presented with ~3% TBSA deep second-degree chemical burns on the surface of the forearm and lower limbs. The skin was raw, macerated and oozing pus, and topical silver sulfadiazine had been applied to the affected area (Fig. 1). The chest auscultation breath sounds were rough, but no rhonchi or rales were observed. The pulse rate was 88 beats/min at a regular rhythm with no extra heart sounds or murmurs on auscultation of the various valve areas. The abdomen was soft and nontender. The liver and spleen were untouched under the ribs. Neurological examinations and the results of the routine blood and urine tests were normal. The serum urea level was 11.3 mmol/l and the serum creatinine level was 145 μmol/l. Computed tomography (CT) revealed interstitial pneumonitis (Fig. 2). Following admission to the Department of Poisoning and Occupational Disease, the patient underwent an urgent surgical debridement and rinse. The patient was administered with 500 mg methylprednisolone per day by an intravenous drip for three days followed by 200 mg intravenously every day, and five days later the dosage of methylprednisolone was gradually decreased according to the condition of the patient. Cyclophosphamide (800 mg) was administered intravenously on the first day and etanercept (25 mg) was administered by a hypodermic injection twice a week for three weeks. Simultaneously, antibiotics and nutrition were actively administered. By the time of discharge, 21 days later, the patient had recovered well. The burns on the limbs had healed, with pigmentation and topical scar formation, kidney function had recovered and the lung CT revealed no signs of pulmonary fibrosis. A month later, the patient presented at the hospital with herpes zoster on the right side of the back and waist. The patient received vitamins B1 and B12 and antiviral therapy and recovered 20 days later.

### Case 2

**Figure 3:**
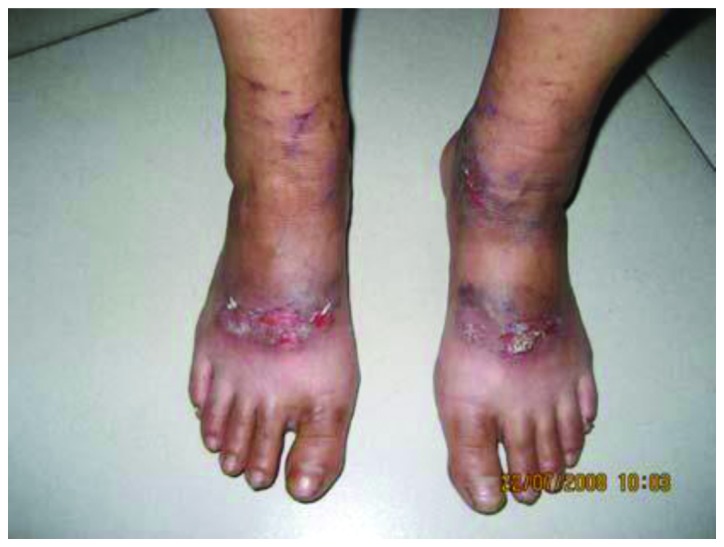
Skin injury caused by paraquat in Case 2.

A 55-year-old female presented at the Qilu Hospital of Shandong University with 2% TBSA shallow second-degree chemical burns on the lower limbs. The patient was a contractual worker spraying paraquat in the orchard with the patient from Case 1 and had also worked from 9:00 am to 12:00 am. Due to the high temperature the upper and lower limbs of the patient were fully exposed to the solution without any protection. Some solution adhered to the skin of the patient, particularly on the lower limbs. The patient did not wash the skin immediately. In the afternoon, the skin coated by paraquat had developed erythema followed by blistering. The patient was treated by a community doctor for five days. Six days subsequent to the incident, the condition of the patient deteriorated and the patient was sent to Qilu Hospital of Shandong University. Upon examination, the mentation of the patient was intact and the mental status was normal. Physically, the patient had 2% TBSA shallow second-degree chemical burns on the forearm and the surface of the lower limbs. The skin was raw, macerated and oozing pus (Fig. 3). The heart, lungs and abdomen were normal. The results of the routine blood tests and liver and kidney functions were normal. The lung CT was also normal. Following admission to the Qilu Hospital of Shandong University, the patient underwent urgent surgical debridement. Methylprednisolone and Levofloxacin (400mg ivdrip per day) were administered for 7 days. Fifteen days later, the burns on the limbs of the patient had healed with pigmentation formation.

## Discussion

Paraquat is a commonly used non-selective herbicide (12). According to the International Program for Chemical Safety Classification System of Pesticides by the World Health Organization (WHO), paraquat is classified as ‘moderately hazardous’ (WHO class II) (13). The herbicide industry claims that paraquat is safe if handled as instructed, yet paraquat poisoning remains a severe health problem globally and the degree of the severity depends on the exposure route and dose. In general, oral ingestion of paraquat is fatal as it is acutely toxic to humans. As a result, many accidental and suicidal deaths have been reported (14–16). In 1978, a middle-aged female was reported to have died from respiratory failure caused by percutaneous paraquat absorption (7). This showed the extreme toxicity of paraquat and demonstrated that lethal quantities may be absorbed from apparently trivial skin wounds. In 1983, it was reported that a patient whose scrotal skin had been exposed to a concentrated paraquat solution suffered from renal and respiratory failure and hepatic damage, although the patient eventually recovered. This demonstrated that dermal exposure to paraquat, particularly via the scrotum, may produce serious systemic toxicity (9). Data from the Israel Poison Information Center regarding 15 cases of single exposure to the skin or eyes during work with paraquat solutions revealed that a single exposure of healthy skin to paraquat solutions caused local lesions. No systemic effect was detected in these patients (17). However, two males from Israel were admitted to hospital for severe pain due to extensive chemical burns in the perineal and scrotal regions, caused by Ducatalon (a mixture of diquat and paraquat) leaking from defective equipment used for spraying the herbicide (18). There has been a recent rise in case reports regarding paraquat poisoning following dermal exposure (19–21). In the two cases documented in this case report, the high temperature and humidity together with the lack of protection for the sprayers increased the risk of dermal exposure. In Case 1, the paraquat entered the body through the damaged skin and caused renal injury and pulmonary fibrosis. In Case 2, the paraquat caused serious skin injuries similar to that of Case 1.

One of our previous studies demonstrated that methylprednisolone combined with cyclophosphamide and intensive etanercept therapy has a curative effect on acute paraquat poisoning (22). Methythylprednisolone, cyclophosphamide and etanercept were used in Case 1 and demonstrated a favorable curative effect. The occurrence of herpes zoster may be associated with the administration of immunosuppressive treatment. The patients eventually recovered fully without any sequelae.

These cases suggest that paraquat is well absorbed through abraded or injured skin and may result in severe toxicity. Therefore, it is important to wash the contaminated skin, including hair and nails, vigorously, with soap following dermal exposure to paraquat. Broken skin should be treated for superficial burns following a thorough cleansing. Stricter precautions, including the mandatory use of protective clothing, should be recommended whenever paraquat is used.
